# Commentator Discussion: Robotic hand-sewn anastomosis versus anastomotic connector in beating heart totally endoscopic coronary artery bypass: A propensity-matched study

**DOI:** 10.1016/j.xjse.2025.100046

**Published:** 2025-02-25

See Article page 100042 in the March 2025 issue.

Presenter: Dr Hiroto Kitahara

**Dr Michael Halkos** *(Atlanta, Ga)*. Great. Okay. Congratulations, thank you for having me. I'm Michael Halkos from Emory. And thank you for sending the manuscript well in advance. Dr Balkhy obviously is recognized as the leading expert in totally endoscopic coronary artery bypass (TECAB) and has consistently shown excellent results with this approach. And, because there continues to be slow but gradual adoption with robotic-assisted approaches, the question remains whether TECAB will ever be adopted by other surgeons. This represents sort of the pinnacle of minimally invasive bypass procedure and is certainly the most technically demanding. So, congratulations on the manuscript, and I just have a few questions. I'll ask one at a time and have you answer. For some, Sam may need to help.

**Figure:**
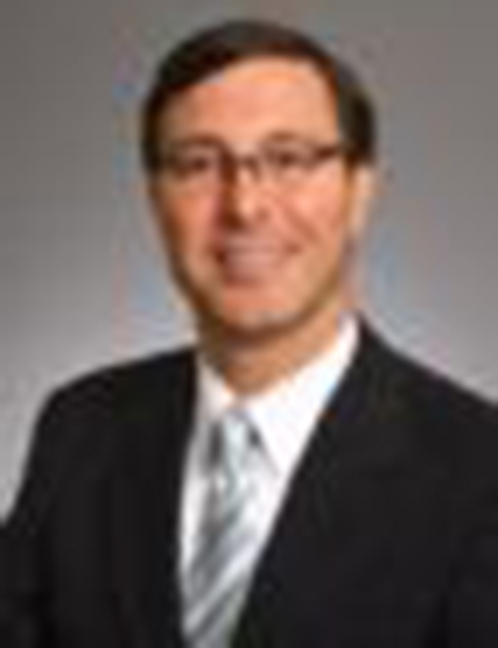

So, even though you had match groups, there were certain anatomical descriptors that were left out in this single-vessel group. Were these patients who just had single-vessel disease with no other disease, or did that include hybrid patients where they had stents to the other side?

**Dr Hiroto Kitahara** *(Chicago, Ill)*. Yeah. Thank you for the question. So, we included the hybrid patients. So, there are several patients who had multivessel disease, which we do the left internal mammary artery left anterior descending artery and then stent on the right coronary artery or the circumflex artery. So, in this group, 20%, 30% of the patients had the hybrid procedure.

**Figure:**
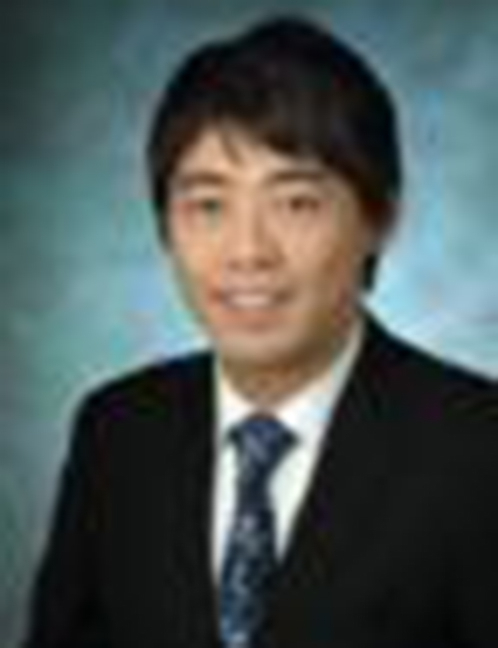

**Dr Halkos**. Pretty balanced in both groups?

**Dr Kitahara**. Yes, it's balanced after matching, even before.

**Dr Halkos**. So, I guess some broader questions, and maybe Sam, so obviously, the anastomotic connector is no longer available and now the SI system may be retiring, and the XI does not have the stabilizer. What's the plan for TECAB in the future? You can try to answer that.

**Dr Kitahara**. Yeah, I can try to answer. I will try. So, I think we are—

**Dr Halkos**. This is what we all want to know because this is what you've pioneered. And what's the future? What's [crosstalk]?

**Dr Kitahara**. We are looking for a way to use it. And XI, first of all, we are looking for company support to invent a different type of stabilizer for the XI use, which solves this problem, I hope, but that takes time. Until then, I think that soon, we are not going to be able to use a stabilizer in XI or SI, so we are going to find the solution. We might use the different company's stabilizer to introduce—

**Dr Halkos**. Which is hours away.

**Dr Kitahara**. —and then on the TECAB setting. So that's the quick solution, but it's difficult to transit to the place where we have a satisfied outcome. But I think he has more explanation.

**Dr Husam H. Balkhy** *(Chicago, Ill)*. No, no. I don't have more, actually. That's a great answer and thank you for the question. But if you want to know the detailed answer, stay until the end of the session because I'm giving a talk on it. [laughter]

**Dr Halkos**. So, given that you have experience, Sam, with both of these, what's your preference? If you had both—if you had the connectors or the sutures?

**Dr Balkhy**. Suturing.

**Dr Halkos**. And are there any other connectors in the pipeline that would be superior to what was previously available, about which a lot of us had reservations?

**Dr Balkhy**. Yeah. So, I'm a huge proponent of devices and automation and quickness, but I must say, and this is in all honesty, we kind of evolve as we go along. I was a huge proponent of this anastomotic device TECAB procedure. I've been sewing now for the last 6 years, and I think sewing the graft is near and dear to a heart surgeon's heart, if you will, and I think that that's what we do best. And so, it's clean, it's open. You saw that the incidence of revision of the anastomosis on the device was 3 in 100 cases and 0 in the other, and I think that speaks to the level of an open anastomosis and how confident you are in your ability to predict that the graft cannot be improved upon when you sew it. Whereas when you do a blind anastomosis with a device, our threshold for revision is fairly low if we have anything less than perfect flow. And we're a little bit spoiled. We're used to flows that are very high, so if we have a low flow that's even in the 30s with a PI not less than 2, we're not happy with that. So, we do some revisions. However, I think in the end, we're used to sewing. What got us to this level is an anastomotic device because that level of the playing field, it allowed us to operate on patients who otherwise may not have wanted to go through a learning curve of sewing beginning in your career. And what happens is when you have a device like this, it gets you through understanding the environment.

The endoscopic environment is a different animal than the open environment. And I think trying to understand where you are, it takes a while just better than most. And so, I think that having the device in the early goings on in my evolution was extremely helpful. To answer your other question, there are 2 companies from the Netherlands that are—they're working on devices. I encourage you all if you're interested in minimally invasive coronary surgery to come to International Society for Minimally Invasive Cardiothoracic Surgery this year. Dr Keyur, our esteemed is giving a talk on anastomotic devices at International Society for Minimally Invasive Cardiothoracic Surgery. And the 2 companies that he's going to be addressing are the AMT, which is a laser anastomotic device. And the University of Utrecht [inaudible] device, the S2, which reminds you of the magnets—back in the day, the Ventrica magnets, but this is actually a clip device. And we've done experimentation with that one in our laboratory. And they both have some promise, but we're still a long way from coming back to that.

**Dr Halkos**. All right. Thank you.

**Dr Balkhy**. Thank you.

**Dr Halkos**. Thank you for your talk.

**Unidentified Speaker 2**. I have a couple of questions for you. And number one is, what do you think is the future—maybe in the future as surgeon, we can lose the opportunity I believe to construe the coronary arteries anastomosis. The second question is, in your place do you use any device to measure the flows between the 2 techniques?

**Dr Kitahara**. Also, the flow measurement we use [inaudible] time for [inaudible] during this intraoperatively and we show that without—and, sorry, what's the first question?

**Unidentified Speaker 2**. The first question is, what do you think about—your opinion in the future maybe that surgeons can lose the opportunity to construe it—and kind of differing skills in the coronary anastomosis when you replace with this technology? Is it a positive impact or a negative impact maybe?

**Dr Kitahara**. Sorry, speak more—?

**Dr Halkos**. Your question is, is there going to be a negative impact on surgeons in the future being able to learn how to do coronary anastomosis? Is that your question?

**Unidentified Speaker 2**. Yes, differing skills about this.

**Dr Kitahara**. With this technique? I don't think so. This is a more advanced technique, so we need to always learn how to do coronary anastomosis, unless we have a special device that is very simple and quick, very reliable, and the best anastomotic connector, easy to use in that situation, we don't need to learn coronary anastomosis, which I don't think it's going to happen.

**Unidentified Speaker 2**. Okay. Thank you.

**Dr Kitahara**. Thank you.

**Unidentified Speaker 3**. Did you catheterize a lot of these patients afterwards? The multivessel stenting seems like an easy opportunity. Do you routinely do that? Do you have a protocol for that? And the ones that you did with the anastomotic device, which is hand-sewn, did you notice any subtle difference? The clips you'll see. But the actual anastomotic connection, could you tell looking at the catheter?

**Dr Kitahara**. We only do the catheter for the patient who has the hybrid procedure after the percutaneous coronary intervention or someone who needs a catheter like amp-1 catheter. And then we see the coronary angiogram for all patients, but actually I found it's bigger than the normal coronary anastomosis, but it's almost the same as the suture technique compared to anastomotic connector. They're much different, I saw, in the catheter.

**Unidentified Speaker 3**. You think they did look different?

**Dr Kitahara**. No, no. They didn't? Yeah.

**Unidentified Speaker 3**. They look pretty similar?

**Dr Kitahara**. Yeah.

**Unidentified Speaker 3**. So, between hand-sewn and anastomotic device, they look about the same?

**Dr Kitahara**. Mm-hmm.

**Unidentified Speaker 3**. That's impressive. Too bad it's gone.

**Unidentified Speaker 4**. Thank you for the great presentation. Do you have any data regarding long-term patency in your study cohort?

**Dr Kitahara**. So, thank you for the question. So, we don't have a longer term in this specific study because since we started this robotic hand-sewn anastomosis from 2018, so it's only 5 years until then. So we need to follow-up the patient group more longer and then maybe 3, 4 years later, we have some—and also, the follow-up not perfect because, I mentioned, we only follow up the hybrid or cath.

**Unidentified Speaker 4**. You mean with catheter examination or computed tomography angiography in your follow-up?

**Dr Kitahara**. Maybe we will do the coronary catheter. I don't do computed tomography angiogram unless clinically necessary.

**Unidentified Speaker 4**. Okay. Thank you.

**Dr Halkos**. One last question and then our moderator but go ahead. Please, go ahead.

**Unidentified Speaker 5**. Thank you so much for the great presentation. I just have one question. So, in this study, you focus on single-vessel disease. So, if you include other patients who have other diseases like right coronary artery or posterior descending artery or circumflex, how do you think about the best approach for the other anastomosis? If you use a connector, is there any anatomical restriction due to the kind of angle from the port, or hand-sewn is better?

**Dr** **Kitahara**. Thank you for the question. So, we actually use this device predominately for all anastomosis before 2018, for any left internal mammary artery left anterior descending artery or right coronary artery or posterior descending or left circumflex. The only difficulty is the coronary artery quality. It's so calcified region the only place where—the one pinpoint place we can anastomosis, in that situation as I showed you the video that the device needs some length of the anastomosis so that in that situation, hand-sewn anastomosis is the only way. So even before 2018, we did hand-sewn anastomosis. But mainly, the normal coronary artery disease, we do this anastomotic connector for almost all patients before.

**Unidentified Speaker 5**. Thanks so much.

**Dr Halkos**. Dr Kitahara, thank you very much for—

**Dr Kitahara**. Thank you.

**Dr Halkos**. —a great presentation.

[applause]

## Conflict of Interest Statement

Dr Balkhy has disclosed that he is a proctor for Intuitive Surgical. All other authors reported no conflicts of interest.

The *Journal* policy requires editors and reviewers to disclose conflicts of interest and to decline handling or reviewing manuscripts for which they may have a conflict of interest. The editors and reviewers of this article have no conflicts of interest.

